# Creation of mutant mice with megabase-sized deletions containing custom-designed breakpoints by means of the CRISPR/Cas9 system

**DOI:** 10.1038/s41598-017-00140-9

**Published:** 2017-03-03

**Authors:** Tomoko Kato, Satoshi Hara, Yuji Goto, Yuya Ogawa, Haruka Okayasu, Souichirou Kubota, Moe Tamano, Miho Terao, Shuji Takada

**Affiliations:** 1 Department of Systems BioMedicine, National Research Institute for Child Health and Development, Tokyo, 157-8535 Japan; 2 Department of Biology, Faculty of Science, Toho University, Miyama 2-2-1, Funabashi, Chiba 274-8510 Japan

## Abstract

The clustered regularly interspaced short palindromic repeat (CRISPR)/CRISPR-associated protein 9 (Cas9) system is a useful tool for creation of mutant mice with mutations mirroring those in human patients. Various methods have been developed for this purpose, including deletions, inversions, and translocations. So far, mutant mice with deletions of up to 1.2 megabases (Mb) have been generated by microinjection of the CRISPR/Cas9 system into fertilized eggs; however, a method for generation of mutant mice with a deletion of more than several Mb size is necessary because such deletions have often been identified as possible causes of human diseases. With an aim to enable the generation of disease models carrying large deletions with a breakpoint in custom-designed sequences, we developed a method for induction of an Mb-sized deletion by microinjection of a pair of sgRNAs, Cas9, and a donor plasmid into fertilized eggs. Using this method, we efficiently and rapidly generated mutant mice carrying deletions up to 5 Mb.

## Introduction

With the rise of genome editing technologies, *i*.*e*., zinc finger nuclease (ZFN)^1^, transcription activator-like effector nuclease (TALEN)^2^, and RNA-guided clustered regularly interspaced short palindrome repeat-associated Cas9 nuclease (CRISPR/Cas9)^3, 4^, creation of mutant mice has become considerably easier. Especially, CRISPR/Cas9 is most widely used, owing to its high efficiency of genome editing and ease of construction of required materials. CRISPR/Cas9 requires a single guide RNA (sgRNA), which recognizes the target sequence, and the Cas9 nuclease, which recognizes the protospacer adjacent motif (PAM) sequence and sgRNA. The Cas9/sgRNA complex induces a double-strand break (DSB) and a mutation is created when the lesion thus generated is repaired. There are mainly two types of repair mechanisms for DSBs. One is nonhomologous end joining (NHEJ), which directly connects two ends and which is known as an error-prone mechanism. The other is homology-directed repair (HDR), in which DSB is repaired by exchange of homologous sequences.

The genome editing technology brings innovation to the design of mouse models of diseases with mutations similar to those found in patients, because this technology facilitates the introduction of precise mutations at a single-nucleotide level, which has been difficult to achieve by traditional methods utilizing HDR of embryonic stem (ES) cells, such as single-nucleotide substitution^5^ and knockout of Y-linked genes^6, 7^. Generation of mutant mice by means of the CRISPR/Cas9 system is performed via microinjection of *in vitro* transcribed sgRNA and Cas9 (mRNA or protein) into fertilized eggs^5–13^. Similarly, chromosomal rearrangements, such as a large deletion^9–13^, inversion^9, 11, 13^, and duplication^13^, can also be induced by microinjection of Cas9 and a pair of sgRNAs, which recognize both ends of the target locus, into zygotes. Actually, there have been several reports on the production of mutant mice with a large deletion allele. Although a 30-kb deletion^9^, a 95-kb deletion^10^, a 0.5-Mb deletion^11^, a 1.15-Mb deletion^12^, and a 1.2-Mb deletion^13^ have been successfully generated by this method so far, the production efficiency of such large deletion alleles with the expected breakpoint is not very high because the DSB ends flanking the deletion target are randomly joined by NHEJ. Recently, a 1.15-Mb deletion with a breakpoint sequence designed for HDR was efficiently induced by microinjection of Cas9 mRNA, a pair of sgRNAs, and donor single-stranded oligodeoxynucleotide (ssODN) containing a breakpoint sequence into fertilized eggs^13^. However, a method to induce a deletion of several Mb in mice is still required to mimic the disease-causing deletions found in human patients in a model animal because the identification of disease-causing deletions has become considerably easier, owing to the development of comparative genomic hybridization technology^14–16^. Previously, a mutant mouse with a deletion of a 1.1-Mb region was generated using an ES cell-based method to mirror a mutation found in a patient with epilepsy, cleft palate, and developmental defects, having a deletion of a 1.1-Mb region in human chromosome Xq22.1^17^. In general, this kind of experiment is time-consuming, laborious, and costly. To enable generation of mutant mice with a deletion of several Mb with a designed breakpoint sequence at a single-nucleotide level, we developed a method based on the CRISPR/Cas9 system. This method enables us to design a breakpoint at a single-nucleotide level.

## Results

### Large deletions can be induced by means of the CRISPR/Cas9 system using a donor plasmid

**Figure 1:**
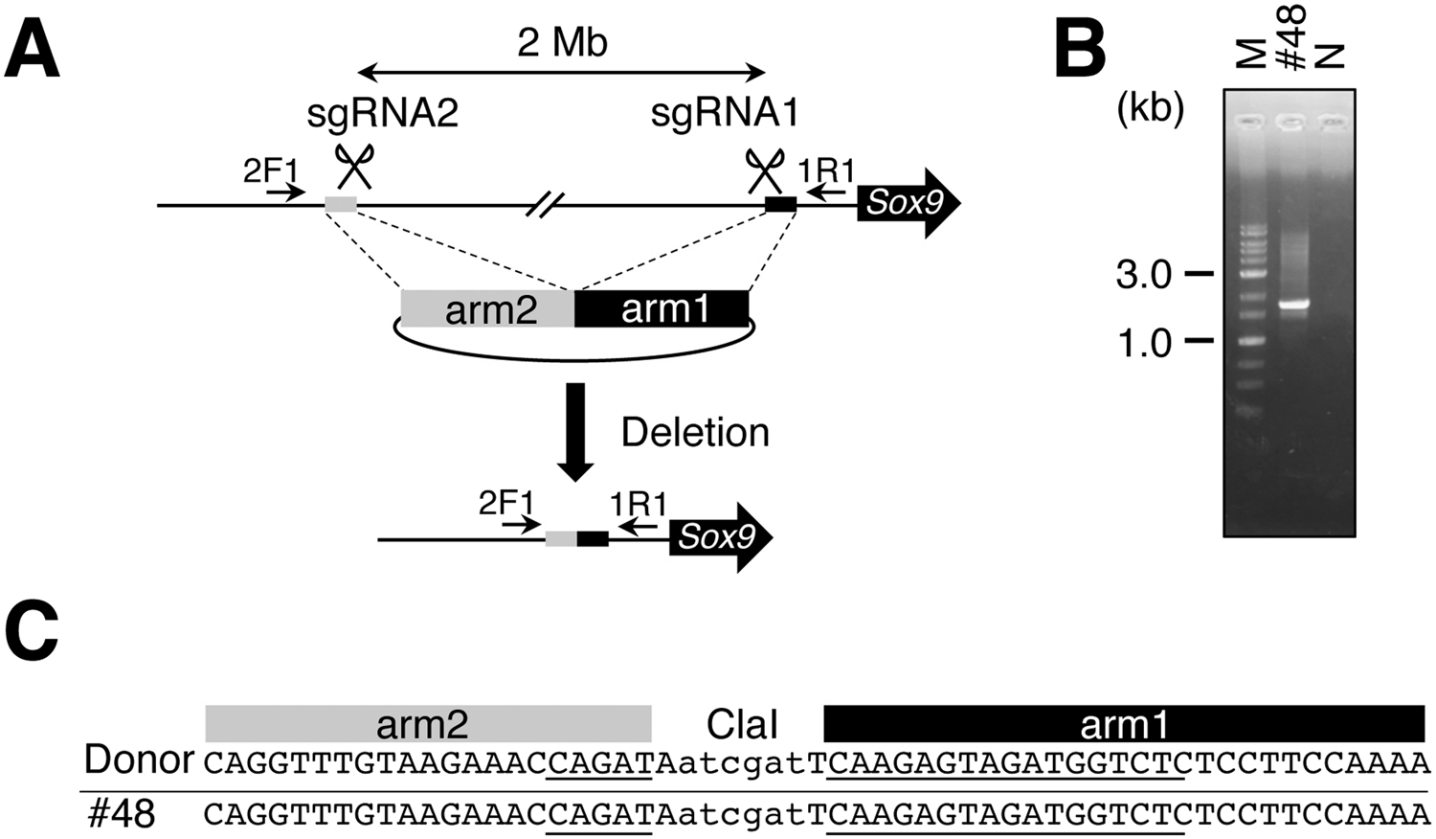
Creation of mutant mice with a 2-Mb deletion with a donor plasmid. (**A**) Schematic representation of the 2-Mb region upstream of *Sox9*. The black line represents genome sequence. Homology arms are indicated with black and gray boxes. PCR primers are indicated with arrows. Positions of sgRNA recognition sequences are indicated with scissors. (**B**) An agarose gel electrophoretogram image of PCR products amplified using 2F1 and 1R1 primers and DNA prepared from #48 embryo. M: a 1-kb DNA ladder (molecular weight markers); N: no template control. (**C**) Sequence of the deletion junction. Sequences of the donor plasmid and of #48 embryo are aligned. Positions corresponding to arms and the ClaI recognition sequence are indicated at the top. Parts of ﻿gRNA recognition sequences are underlined. Black and gray boxes correspond to those of (**A**).

Aida *et al*. showed that knock-in mice could be efficiently generated using CRISPR/Cas9 with a donor plasmid when Cas9 protein is introduced into fertilized eggs^8^. This prompted us to test whether mutant mice with large deletions (Mb size) would be generated efficiently by their method. To test this possibility, a pair of sgRNAs that recognize upstream sequence of the *Sox9* gene on chromosome 11, the Cas9 protein, and a circular plasmid (containing a pair of arms of 1 kb each with a part of sgRNA sequence at one end of each arm as a donor (Fig. 1A), so that the arms could not be recognized by sgRNAs) were injected into the pronucleus of fertilized eggs. The embryos were transferred to the oviduct of pseudopregnant female mice and collected 10 days after the transfer because the deletion target was set on the regulatory sequence of *Sox9* gene, whose deletion can cause embryonic death after embryonic day 11^18^. When sgRNA1 and sgRNA2 (designed to be 2 Mb apart), Cas9 protein, and a donor circular plasmid containing arm2 and arm1 were injected, one out of 40 embryos was identified as a mutant carrying the desired deletion according to agarose gel electrophoresis of genotyping PCR products obtained by means of primers 2F1 and 1R1, which were designed to match the genome sequence outside arm1 and arm2 (Fig. 1B, Table 1). Sequence analysis of the PCR product showed that the sequence was the same as that of the donor sequence including the junction of arm2 and arm1, suggesting that the deletion was introduced by HDR (Fig. 1C, Supplementary Figure 1). To determine whether the deletion occurred homozygously in all cells, we PCR-amplified genomic regions around sgRNA targets using primer pairs 1F3/1R1 and 2F2/2R2. PCR products were obtained from both regions (Supplementary Figure 4A,B), suggesting that not all cells contained the deletion homozygously.

**Table 1:** Summary of microinjection-based generation of mutant mice.

sgRNA combination	Donor	No. of injected eggs/2-cell embryos	No. of transferred embryos	No. of genotyped embryos	Deletion				Inversion	
					Total	HDR mechanism	NHEJ mechanism	Unknown mechanism	One breakpoint mapped	Two breakpoints mapped
sgRNA1/sgRNA2	Plasmid	195/131	120	40	1 (2.5)	1 (100)	0	0	0	0
sgRNA1/sgRNA2	—	111/100	36	12	0	N/A	0	0	0	0
sgRNA1/sgRNA2	—	84/75	75	39	0	N/A	0	0	0	0
sgRNA1/sgRNA3	Plasmid	152/132	120	37	7 (19)	3 (43)	4 (57)	0	1 (2.7)	1 (2.7)
sgRNA1/sgRNA3	ssODN	200/165	165	42	11 (26)	5 (45)	1 (9)	5 (45)	1 (2.4)	4 (9.5)
sgRNA1/sgRNA3	—	88/66	66	23	1 (4.3)	N/A	1 (100)	0	1 (4.3)	0 (0)
sgRNA1/sgRNA3	—	43/40	40	23	1 (4.3)	N/A	1 (100)	0	1 (4.3)	1 (4.3)

The numbers in parentheses are Total, One, and Two breakpoints mapped representing the percentages calculated from the number of mutants relative to the number of genotyped embryos. The numbers in parentheses in HDR mechanism, NHEJ mechanism, or Unknown mechanism represent the percentages calculated from the number of mutants relative to the number of total deletions. The dash means that no donor was used; N/A: not applicable.

**Figure 2:**
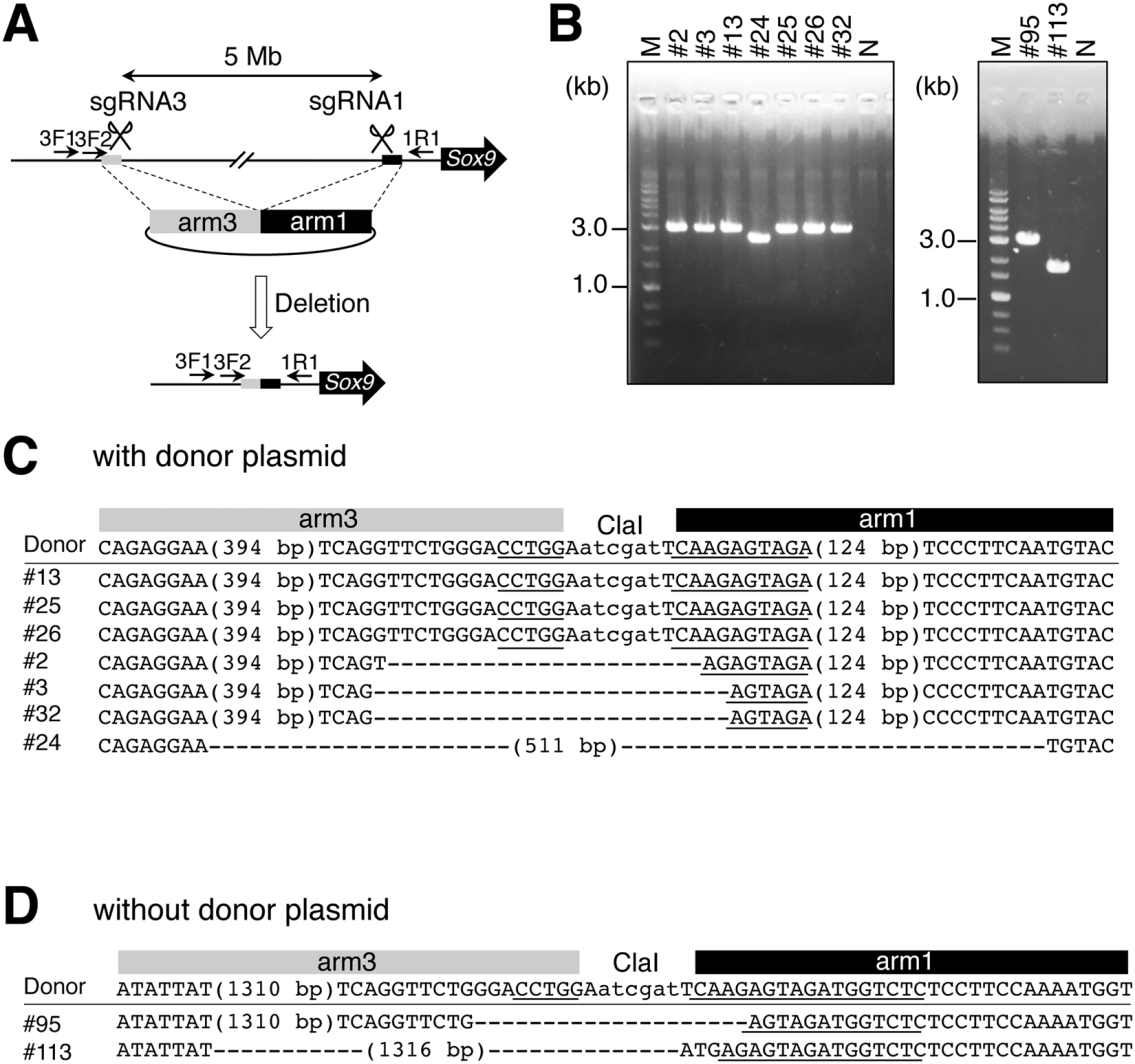
Generation of mutant mice with a 5-Mb deletion with or without a donor plasmid. (**A**) Schematic representation of the 5-Mb region upstream of *Sox9*. The black line represents a genome sequence. Homology arms in a donor plasmid are indicated with black and gray boxes. PCR primers are indicated with arrows. Positions of sgRNA recognition sequences are denoted with scissors. (**B**) An agarose gel electrophoretogram image of PCR products amplified using 3F1 and 1R1 primers and DNA prepared from embryos injected with the donor plasmid (left panel) and without the donor plasmid (right panel). Embryo IDs are shown above images with the # sign. M: a 1-kb DNA ladder (markers); N: no template control. (**C**) Sequences of the donor plasmid and of embryos injected with the donor plasmid are aligned. Positions corresponding to arms and ClaI recognition sequence are indicated at the top. (**D**) Sequences of the donor plasmid and of embryos injected without the donor plasmid are aligned. Positions corresponding to arms and ClaI recognition sequence are indicated at the top. Parts of gRNA recognition sequences are underlined. Black and gray boxes correspond to those in (**A**).

To test whether greater than 2-Mb deletions can be induced by this method, we tried to generate mutant mice with a 5-Mb deletion using sgRNA1, sgRNA3, Cas9 protein, and a donor circular plasmid containing arm3 and arm1. Agarose gel electrophoresis of genotyping PCR with primers 3F1 and 1R1, both of which were designed to match the genome outside target sequences arm3 and arm1, revealed that seven out of 37 embryos were deletion mutants (Fig. 2B, Table 1). Sequencing analysis of the PCR products showed that three carried the same sequence as that of the donor sequence, including the junction of arm3 and arm1 (#13, #25, #26 in Fig. 2C, Supplementary Figure 2), whereas the remaining four contained sequences of arm3 and arm1; however, sequences of the junction were different from that of the donor plasmid (#2, #3, #32, #24 in Fig. 2C, Supplementary Figure 2), indicating that the former three were generated by HDR and the latter by NHEJ. To determine whether the deletions occurred homozygously in all cells, we PCR-amplified genomic regions around sgRNA targets using primer pairs 1F3/1R1 and 3F1/3R2. The PCR products were obtained from both regions in all samples (Supplementary Figure 4A,C), suggesting that not all cells contained the homozygous deletion in all samples.

### Large deletions can be created by the CRISPR/Cas9 system without a donor plasmid

To test whether a donor plasmid is necessary to create large deletions, we next tried to make mutant mice with large deletions using the same method as above, but without the donor plasmid. By microinjection of sgRNA1, sgRNA2, and the Cas9 protein into the pronucleus of fertilized eggs, 51 embryos (12 at first try and 39 at second try) were obtained, but no embryo was identified as a mutant with a 2-Mb deletion (Table 1). By microinjection of sgRNA1, sgRNA3, and the Cas9 protein, 46 embryos (23 at first try and 23 at second try) were obtained (Table 1). Agarose gel electrophoresis of genotyping PCR using primers 3F1 and 1R1 showed that two embryos among them had a 5-Mb deletion (Fig. 2B, Table 1). Sequencing analysis of the PCR products showed that the 5-Mb deletions were actually generated, (#95, #113 of Fig. 2D, Supplementary Figure 2) possibly by NHEJ. To determine whether the deletions occurred homozygously in all cells, we PCR-amplified genomic regions around sgRNA targets using primer pairs 1F3/1R1 and 3F1/3R2. PCR products were obtained from both regions in all samples (Supplementary Figure 4A,C), suggesting that not all cells contained the homozygous deletion in all samples.

### Large deletions can be induced by the CRISPR/Cas9 system using an ssODN template

**Figure 3:**
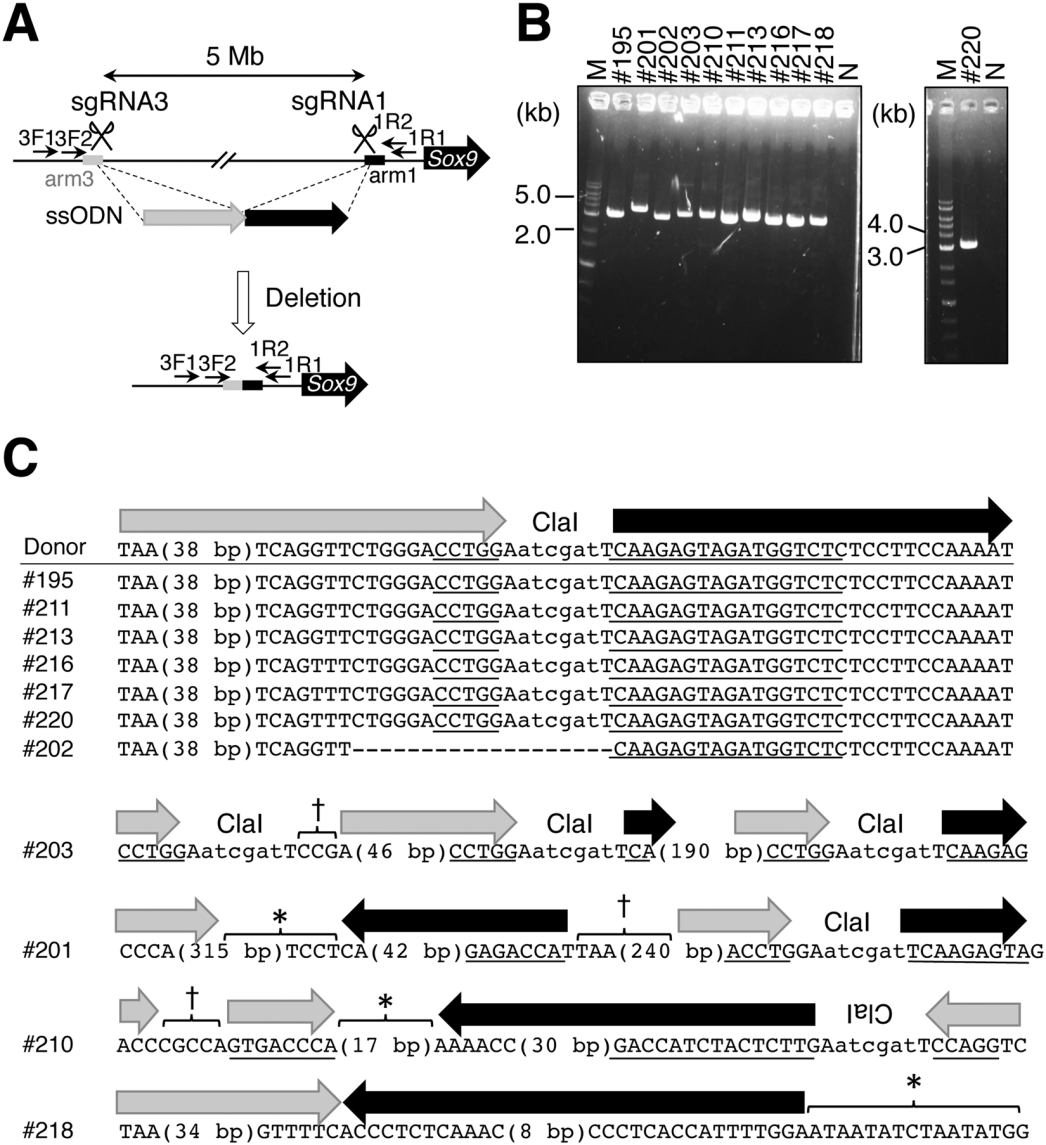
Generation of mutant mice with a 5-Mb deletion using an ssODN template. (**A**) Schematic representation of the 5-Mb region upstream of *Sox9*. The black line represents genome sequence. Homology arms in the ssODN are indicated with black and gray boxes. PCR primers are indicated with arrows. Positions of sgRNA recognition sequences are denoted with scissors. (**B**) An agarose gel electrophoretogram image of PCR products amplified using 3F1 and 1R1 primers and DNA prepared from embryos injected with the ssODN template. Embryo IDs are shown above images with the # sign. M: a 1-kb DNA ladder (markers); N: no template control. (**C**) Sequences of the ssODN template and of embryos injected with the ssODN template are aligned. Positions corresponding to arms and ClaI recognition sequence are indicated at the top. Parts of gRNA recognition sequences are underlined. The asterisk indicates an unknown sequence. The obelisk indicates genomic sequence nonhomologous to the ssODN or to the internal sequence flanked by sgRNA3 and sgRNA1. Black and gray arrows correspond to those in (**A**).

Next, we attempted to induce a 5-Mb deletion by microinjection of the Cas9 protein, sgRNA1, sgRNA3, and an ssODN template, which had a ClaI site flanked by 60 nucleotides bearing homology to upstream and downstream sequences of the target sequence (Fig. 3A) into the pronucleus of fertilized eggs. Eleven out of 42 embryos were found to be mutants by agarose gel electrophoresis after PCR with primers 3F1 and 1R1 (Fig. 3B, Table 1). Sequencing analysis of the amplicons showed that six contained the same sequence as that of the ssODN template at the breakpoint generated by HDR repair (## 195, 211, 213, 216, 217, 220 in Fig. 3C, Supplementary Figure 2) and that a breakpoint in one embryo was joined around gRNA sequences, which were different from the ssODN sequence, possibly generated by NHEJ (#202 of Fig. 3C, Supplementary Figure 2). The remaining five embryos had complex breakpoint sequences (## 201, 203, 210, and 218 in Fig. 3C, Supplementary Figure 2, Table 1). Although the exact reason remains unknown, these mutants may have been generated by HDR, at least in part, in ## 201, 203, 210, because the ClaI recognition sequence, present in the ssODN near the breakpoint region, was identified in these embryos. To find out whether the deletions occurred homozygously in all cells, we PCR-amplified genomic regions around sgRNA targets using primer pairs 1F3/1R1 and 3F1/3R2. PCR products were obtained from both regions in all samples (Supplementary Figure 4A,C), suggesting that not all cells contained the homozygous deletion in all samples.

### Large inversions can be induced by the CRISPR/Cas9 system

**Figure 4:**
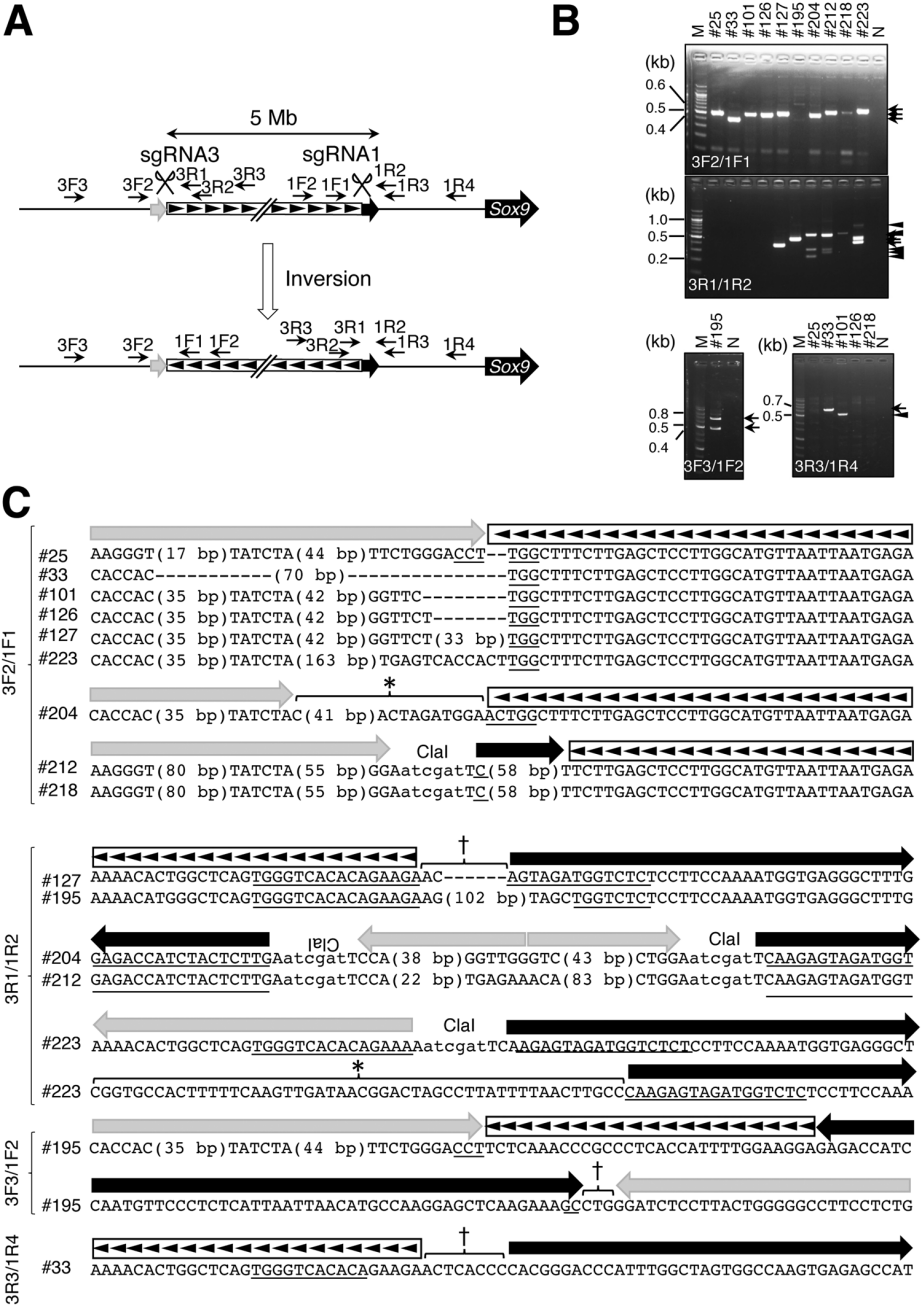
Generation of mutant mice with a 5-Mb inversion. (**A**) Schematic representation of the 5-Mb region upstream of *Sox9*. The black line represents a genome sequence. Homology arms are indicated with black (arm1) and gray (arm3) boxes. PCR primers are indicated with arrows. The inverted region is denoted by a box containing arrowheads. (**B**) Agarose gel electrophoretogram images of PCR products amplified using 3F2/1F1 (upper panel), 3R1/1R2 (middle panel), 3F3/1F2 (lower left panel), and 3R3/1R4 (lower right panel) primer sets and DNA prepared from the embryos. Sample ID is indicated at top of each panel. Arrows and arrowheads indicate positive and nonspecific signals, respectively. M: a 100-bp DNA ladder (markers); N: no-template control. (**C**) Sequences of inversion junctions. Results of direct sequencing of PCR products shown in (**B**). Corresponding sequences of arms and inverted sequences are shown at the top of each panel. Parts of gRNA recognition sequences are underlined. Asterisks indicate an unknown sequence. The obelisk indicates a genomic sequence nonhomologous to the arms or to an internal sequence flanked by sgRNA3 and sgRNA1. Black and gray arrows correspond to those in (**A**). The inverted region is indicated with a box containing arrowheads as shown in (**A**).

Many research groups, including ours, have demonstrated that inversions could be induced when a pair of sgRNAs, which recognizes a *cis* genomic locus, and Cas9 are introduced into the cells^11–13, 19^. Hence, we next tested whether large inversions can be induced in the embryos obtained in the above experiments. In order to identify inversions of 2-Mb regions by screening, PCR was performed using primer pairs 2F1/1F1 and 2R1/1R1 with genomic DNAs purified from embryos injected with sgRNA1, sgRNA2, and Cas9 with or without a donor plasmid as a template. No amplification was observed in any of the embryos, implying that inversion did not occur. Next, the inversion of 5-Mb region was identified by screening with primer pairs 3F2/1F1 and 3R2/1R3 and genomic DNAs purified from embryos injected with sgRNA1, sgRNA3, Cas9 with or without a donor plasmid as a template (Fig. 4A). Since PCR using 3R2/1R3 was not specific for further analysis, nested PCR using the primer pair 3R1/1R2 was carried out (Fig. 4A). Agarose gel electrophoresis of the PCR products obtained by means of 3F2/1F1 and 3R1/1R2 primer pairs revealed that bands of expected sizes were yielded by a total of nine and five embryos, respectively (Fig. 4B, Table 1) and sequence analysis of the PCR products showed that they all contained inversion breakpoints. In #223 amplified with the 3R1/1R2 primer pair, there are two sizes of the PCR products, both contained inversion breakpoints. Since only one end of inversion breakpoints was identified in #195 and #218 using 3R1/1R2 primers and in #25, #33, #101, and #126 using 3F2/1F1 primers, primer pairs 3F3/1F2 and 3R3/1R4 were also used for amplification of the unidentified breakpoints of #195 and #25, #33, #101, #126 and #218, respectively (Fig. 4A). PCR products were identified in #33, #101, and #195. Sequencing analysis of the PCR products showed that a single band of lane #33 and both bands in lane #195 contained inversion breakpoint sequences but that the PCR product of #101 was amplified nonspecifically (Fig. 4B, Supplementary Figure 3). In total, both breakpoints of inversion were identified in six embryos (#33, #127, #195, #204, #212, and #223), while either breakpoint of inversion could not be identified in four embryos (#25, #110, #126, and #218).

To determine whether the inversion occurred homozygously in all cells, we PCR-amplified genomic regions around sgRNA targets using primer pairs 1F3/1R1 and 3F1/3R2. The amplicons were obtained from at least one region in all samples except #223 (Supplementary Figure 4A,C), suggesting that not all cells contained the homozygous inversion in all samples, except #223. There is a possibility that #223 had the inversion homozygously without mosaicism.

In addition, we tried to identify a duplication by PCR using primer pairs 1F3/3R1, 1F3/3R2, 1F1/3R1, 1F1/3R2, 1F3/2R1, 1F3/2R2, 1F1/2R1, and 1F1/2R2, but no PCR product containing the duplication breakpoint was obtained.

### Confirmation of a large deletion by fluorescence *in situ* hybridization (FISH) analysis

**Figure 5:**
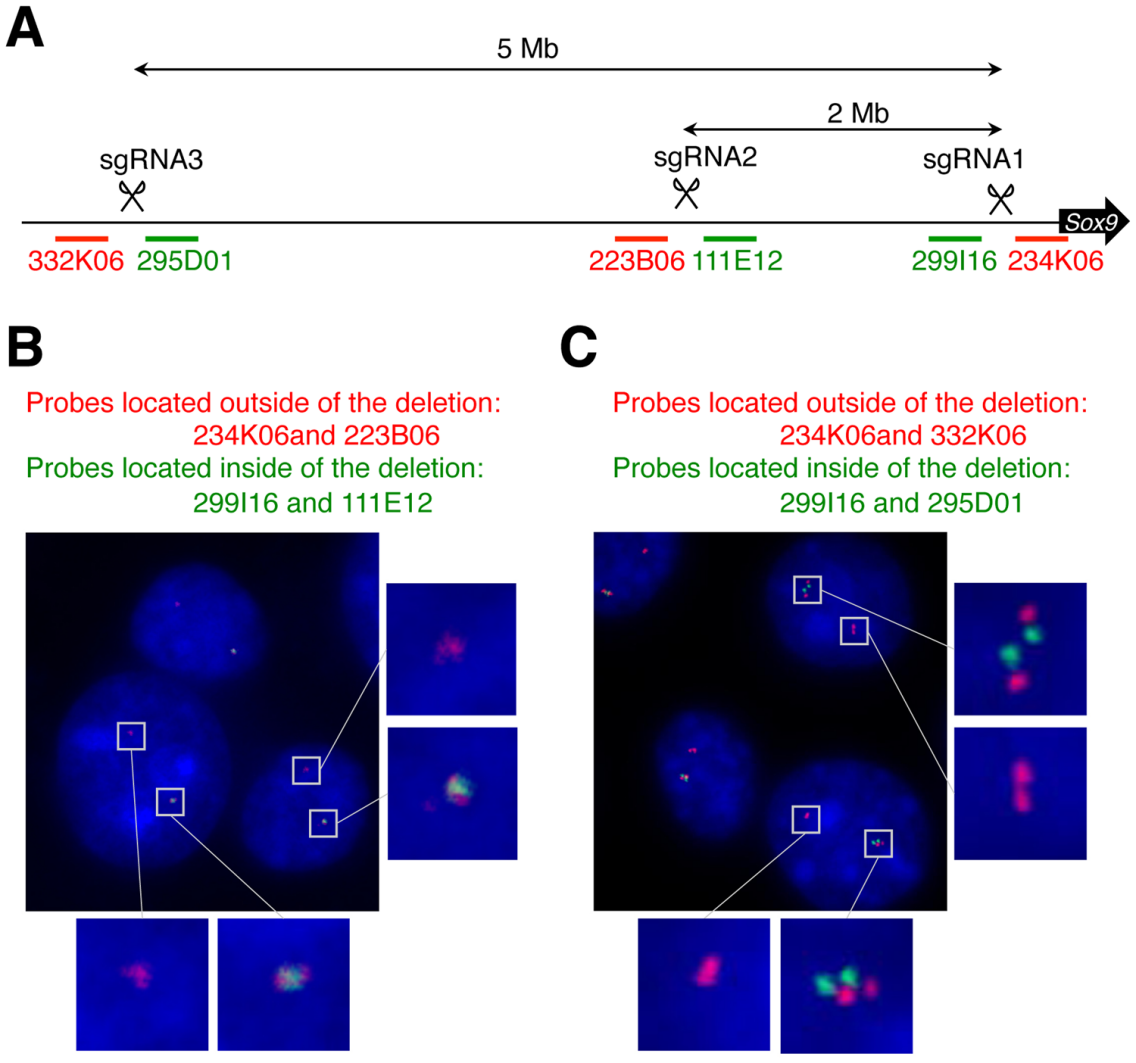
FISH analysis of 2-Mb and 5-Mb deletions. (**A**) Schematic representation of the positions of BAC probes. BACs located within the sgRNAs are shown with green lines and letters, and those outside in red lines and letters. (**B**,**C**) Representative nucleus images of DNA FISH analysis of fibroblasts obtained from #48 embryo of Fig. 1 (**B**, 2-Mb deletion) and #13 embryo of Fig. 2 (**C**, 5-Mb deletion). Probes that we used are indicated at the top of the images with probe colors. Enlarged images of boxed areas are placed on the right.

Deletions of 2-Mb and 5-Mb regions were further confirmed by DNA FISH analysis using fibroblastic cells prepared from embryos identified as mutants with deletion, by PCR analyses of the 2-Mb deletion (#48 shown in Fig. 1) and of the 5-Mb deletion (#13 in Fig. 2). As probes, we used a bacterial artificial chromosome (BAC) harboring inside (B6Ng01-234K06 and B6Ng01-223B06) and outside (B6Ng01-111E12 and B6Ng01-299I16) sgRNA1 and sgRNA2 sequences and inside (B6Ng01-295D01 and B6Ng01-223B06) and outside (B6Ng01-332K06 and B6Ng01-111E12) sgRNA1 and sgRNA3 sequences (Fig. 5A). As a result, two foci were observed in the nuclei of most cells: one had four signals (two pairs of inside outside probes) and the other had a pair of signals of outside probes only, in all the mutants tested. These findings suggest that the embryos contained a 2-Mb or 5-Mb deletion in one chromosome in a cell.

## Discussion

In the present study, we generated mutant mice containing a 5-Mb deletion with a custom-designed breakpoint sequence by microinjection of a pair of sgRNAs, Cas9, protein, and a donor circular plasmid into fertilized eggs. To the best of our knowledge, this is the first report of a method for creation of mutant mice with a Mb-sized deletion using a donor plasmid and the Cas9 protein.

It seems likely that induction of a small deletion is more efficient than that of a large deletion; however, our results indicate that the 5-Mb deletion was induced more efficiently than the 2-Mb deletion. This finding may be associated with differences in sgRNA sequences (efficiencies of genome editing depend on sgRNA sequences), open chromatin conformation around sgRNAs’ targets, or distances between sgRNA targets in the nucleus.

It has been reported that an inversion of 0.5 and 1.15 Mb can be induced by the introduction of a pair of sgRNAs and Cas9^11, 13^. In the present study, we showed that an inversion of 5 Mb can be created. In both cases, efficiencies of induction of the inversion were not high. Although inversion mutant mice could be obtained by increasing the number of microinjected eggs at low efficiency, the efficiency may be increased by adding a pair of donor plasmids to the microinjection solution, each containing sequences around the junction in different orientation.

In some mutant embryos, one end was identified by PCR as inverted using the 3F1/1F1 primer pair, but the other end was not. We cannot come up with the reason behind this phenomenon, but this type of reaction may be more efficient than the inversion reaction occurring at both ends precisely, because we have previously observed similar results when a 0.5-Mb deletion was induced^11^.

One advantage of using a circular plasmid as a donor is that the breakpoint of the deletion can be designed. Without a donor, random lesions are generated by NHEJ. For generation of disease-causing deletions in model mice or induced pluripotent stem (iPS) cells, it is ideal to induce the same breakpoint in models at a single-nucleotide level. Our methods of microinjection including a pair of sgRNAs, Cas9, and a donor circular plasmid in fertilized eggs expand the tool kit for production of animal models of diseases with large deletions by means of a breakpoint custom-designed sequences in their genome.

## Materials and Methods

### sgRNA synthesis

The sgRNA cloning vector was obtained from Addgene (Plasmid #41824). sgRNA sequences were selected using CRISPRdirect (http://crispr.dbcls.jp)^20^. The selected sequences were sgRNA1 (5′-GAGACCATCTACTCTTGCCTTGG-3′), sgRNA2 (5′-AGATAATTACCATGCAACTTGG-3′), and sgRNA3 (5′-GGGTCACACAGAAGACGCCAGG-3′) where proto-spacer adjacent motif (PAM) sequences are underlined. sgRNAs were prepared as previously described^6^. sgRNA sequences except the first nucleotide and PAM were cloned into sgRNA cloning vectors by inverse PCR using primer pairs sgRNA1F/sgRNA1R for sgRNA1, sgRNA2F/sgRNA2R for sgRNA2, and sgRNA3F/sgRNA3R for sgRNA3 (Supplementary Table 1). sgRNA fragments were amplified using T7-added primers (T7sgRNA1F for sgRNA1, T7sgRNA2F for sgRNA2, and T7sgRNA3F for sgRNA3) and sgRNA-R (Supplementary Table 1). sgRNAs were synthesized by means of an *in vitro* transcription reaction using the mMESSAGE mMACHINE T7 Transcription Kit (Ambion). Transcribed RNAs were purified using the MEGAclear RNA Kit (Ambion).

### Donor plasmid construction

Homology arms were amplified by PCR using primer pairs arm1FClaI/arm1RHindIII for arm1, arm2FSalI/arm2RClaI for arm2, and arm3FSalI/arm3RClaI for arm3, and the C57BL/6 genomic DNA as a template (Supplementary Table 1). The amplified fragments were cloned into the pCR-BluntII vector (Thermo Fisher Scientific Inc.). The inserts of arm2 and arm3 were excised by means of SalI/ClaI and subcloned into the pBluescriptII KS vector at HindIII/SalI together with the insert of arm1 excised by ClaI/HindIII. Plasmids were purified using the PureLink HiPure Plasmid Filter Midiprep Kit (Thermo Fisher Scientific Inc.). The purified plasmid was treated with proteinase K (0.5 mg/ml; Roche Diagnostics GmbH) and 0.5% SDS, and then purified by phenol/chloroform extraction and ethanol precipitation to remove RNase completely.

### Microinjection

Fertilized eggs were obtained by mating of superovulated F1 hybrid (C57BL/6 × DBA/2) BDF1 female and male mice with the same genetic background. Superovulation was achieved by standard procedures^21^. A mixture of the Cas9 protein (100 ng/µl; New England Biolabs), sgRNA mix (250 ng/µl each), and donor plasmid (10 ng/µl) or ssODN (100 ng/µl) was microinjected into the pronucleus of fertilized eggs, which were then cultured in the KSOM medium. When a donor plasmid or ssODN was used, the mixture was injected into the pronucleus of fertilized eggs. On the next day, embryos at the two-cell stage were transferred to pseudopregnant ICR female mice. All the mice were obtained from the Sankyo Labo Service Corporation. All animal protocols were approved by the Animal Care and Use Committee of the National Research Institute for Child Health and Development, Tokyo, Japan. All experiments were conducted in accordance with these approved animal protocols.

### Genotyping

Embryos injected with a plasmid were collected at nine or ten days after transfer into a pseudopregnant female, minced, and divided into two groups. One was used for DNA extraction for genotyping and the other was used for fibroblast culture. Embryos injected without the plasmid were collected 10 days after the transfer into the pseudopregnant female and used for DNA extraction for genotyping. Genotypes were determined by PCR and agarose gel electrophoresis, PCR direct sequencing using ExoSAP-IT (USB; Affymetrix) or cloning and sequencing of PCR products using the pGEM-TEasy vector (Promega) and Zero Blunt PCR Cloning Kit (Thermo Fisher Scientific Inc.).

### FISH analysis

Fibroblast cultures were prepared as described elsewhere^11^. Briefly, a minced embryo was sequentially treated with 0.05% trypsin-EDTA (Gibco; Thermo Fisher Scientific Inc.) and 0.1% collagenase (Wako Pure Chemical Industries Ltd.), and then cultured in DMEM (Gibco) supplemented with 10% of FBS (Biological Industries, Cromwell, CT), 1 mM sodium pyruvate (Gibco), 1 mM nonessential amino acids (Gibco), 1% GlutaMAX (Gibco), and 100 µM 2-mercaptoethanol (Sigma-Aldrich, St Louis, MO). Cultured cells were trypsinized, followed by hypotonic treatment with 0.5% KCl and fixation with a 3:1 mixture methanol:acetic acid. Chromosome spreads were prepared by air-drying methods.

As probes, C57BL/6N mouse BAC clones were obtained from the RIKEN BRC through the National Bio-Resource Project of MEXT, Japan. BAC DNAs were purified by NucleoBond BAC 100 (Takara Bio). FISH was performed as described before^11^. The BAC DNAs were digested with EcoRI and labeled by nick translation using the DIG (digoxigenin: for inside) or the BIO (biotin: for outside)-Nick Translation Mix (Roche Diagnostics GmbH). Before hybridization, chromosome spreads were dipped into 70% formamide/2× SSC at 70 °C and dehydrated in ethanol. The slides were hybridized at 42 °C using a hybridization solution (10 µg/ml labeled probes in 50% formamide, 2× SSC, 10% dextran sulfate, 100 µg/ml COT human DNA [Roche], 2 mg/ml BSA) and washed with 2× SSC and 50% formamide in 2× SSC. DIG and biotin were detected by means of 4 µg/ml FITC-conjugated anti-digoxigenin sheep polyclonal antibody (Roche) and 2 µg/ml Alexa 555-conjugated streptavidin (Thermo Fisher Scientific Inc.), respectively, in Tris-buffered saline supplemented with 0.05% of Tween 20. The slides were mounted using ProLong Gold Antifade Mountant with DAPI (Thermo Fisher Scientific Inc.), and images were acquired using a Microscope Axio Imager A2 (Carl Zeiss) at 600× magnification.

## Electronic supplementary material

Supplementary_info
