# Supplementary material for: Creation of mutant mice with megabase-sized deletions containing custom-designed breakpoints by means of the CRISPR/Cas9 system
Source: Sci Rep. 2017 Mar 3;7:59. doi: 10.1038/s41598-017-00140-9 (PMC5427885; doi:10.1038/s41598-017-00140-9)
Supplement: Supplementary file 1 — Supplementary_info [file 41598_2017_140_MOESM1_ESM.pdf]

SUPPLEMENTARY FIGURES AND TABLES

Creation of mutant mice with megabase-sized deletions containing custom-designed breakpoints by means of the CRISPR/Cas9 system

Tomoko Kato,[1,#] Satoshi Hara,[1,#] Yuji Goto,[2] Yuya Ogawa,[1,2] Haruka Okayasu,[1] Souichirou Kubota,[2] Moe Tamano,[1] Miho Terao,[1] and Shuji Takada[1,*]

[1]Department of Systems BioMedicine, National Research Institute for Child Health and Development, Tokyo 157-8535, Japan
[2]Department of Biology, Faculty of Science, Toho University, Miyama 2-2-1, Funabashi, Chiba 274-8510, Japan

[#]These authors contributed equally to this work.
*Correspondence should be addressed to: S.T. (takada-s@ncchd.go.jp, 2-10-1 Okura, Setagaya, Tokyo 157-8535, Japan, Tel. & Fax: +81 3-3417-2498).

Supplementary Figure 1.

#48
(2F1)

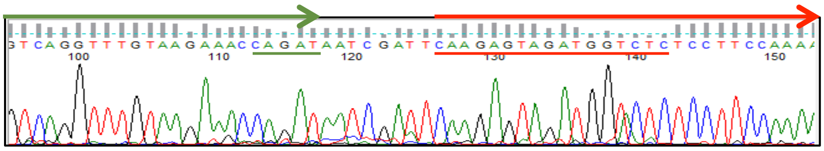

Supplementary Figure 1. Sequence of a 2-Mb deletion junction.
An electrophoretogram of a PCR product amplified using primers 2F1/1R1. The arm2 and arm1 regions are indicated with green and red arrows, respectively. sgRNA2 and sgRNA1 are highlighted with green and red lines, respectively. The word 2F1 in parentheses under the embryo ID represents the primer used for sequencing.

Supplementary Figure 2.

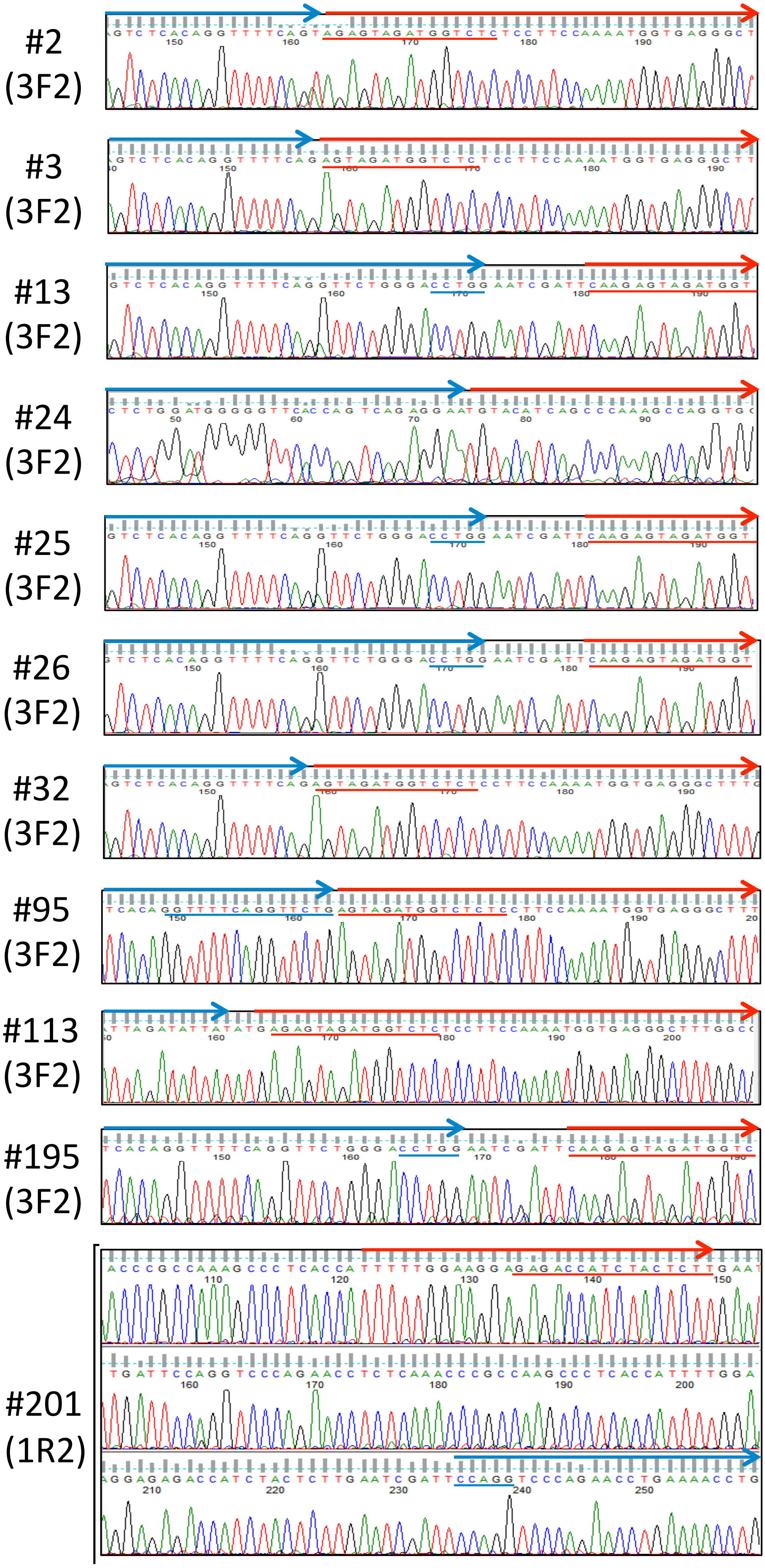

Supplementary Figure 2. continue

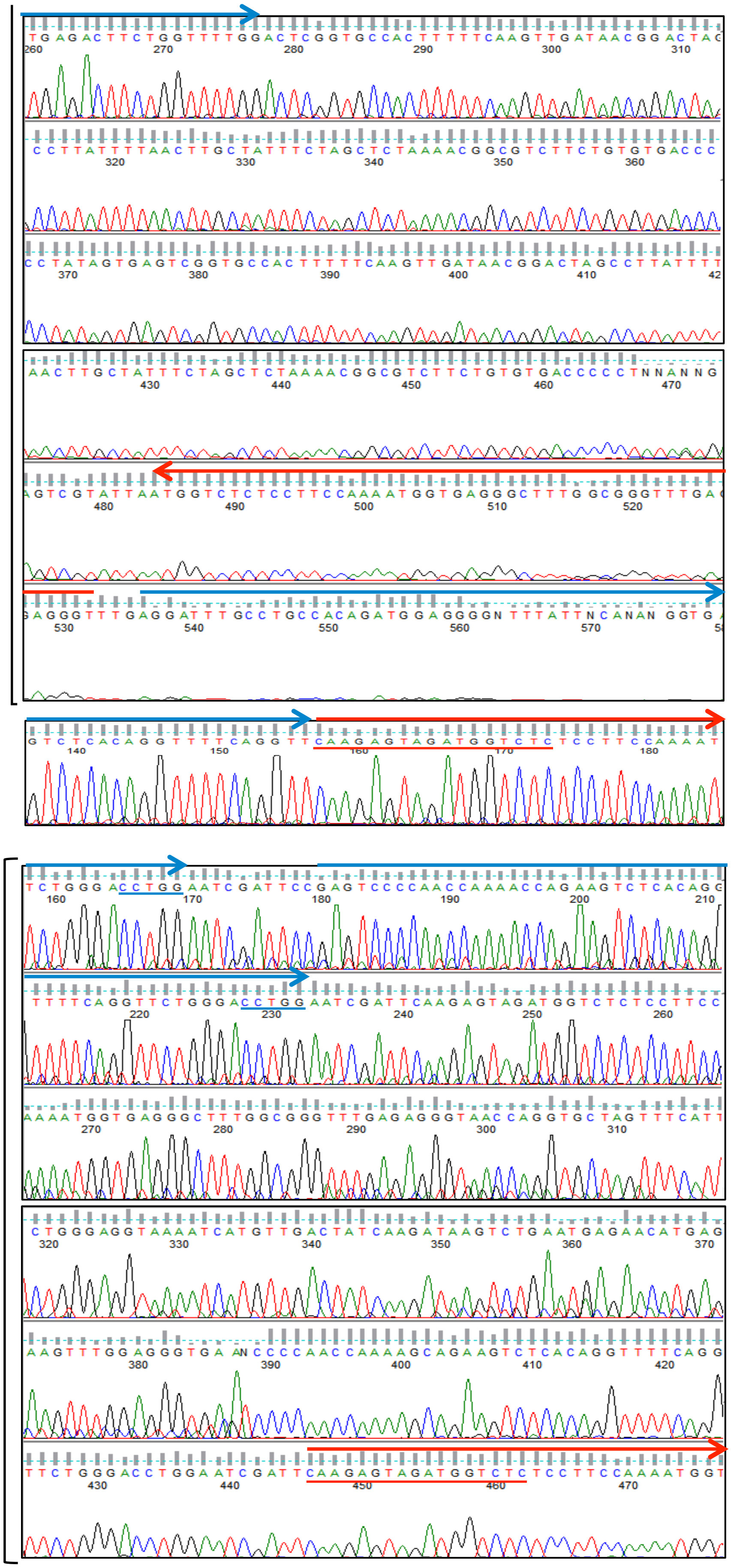

#202 (3F2)

#203 (3F2)

Supplementary Figure 2. continue

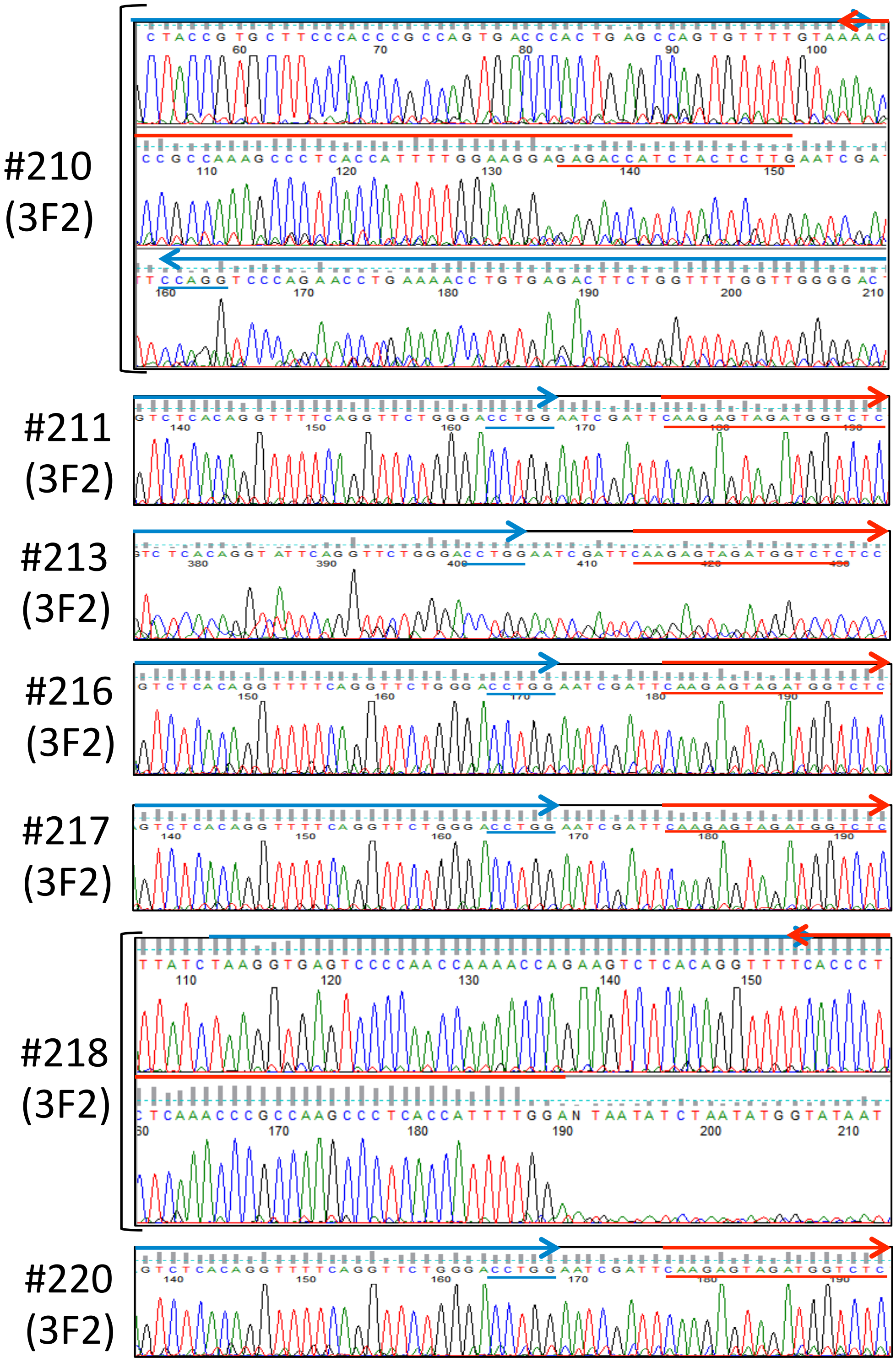

Supplementary Figure 2. Sequence of a 5-Mb deletion junction .
Electrophoretogram of PCR products amplified with primers 3F1/1R1. Sequences arm3 and arm1 are indicated with blue and red arrows, respectively. sgRNA3 and sgRNA1 are indicated with blue and red lines, respectively. Words 3F2 and 1R2 in parentheses under the embryo ID represent the primers used for sequencing.

Supplementary Figure 3.

(3F1/1F1: detecting 5’ junction)

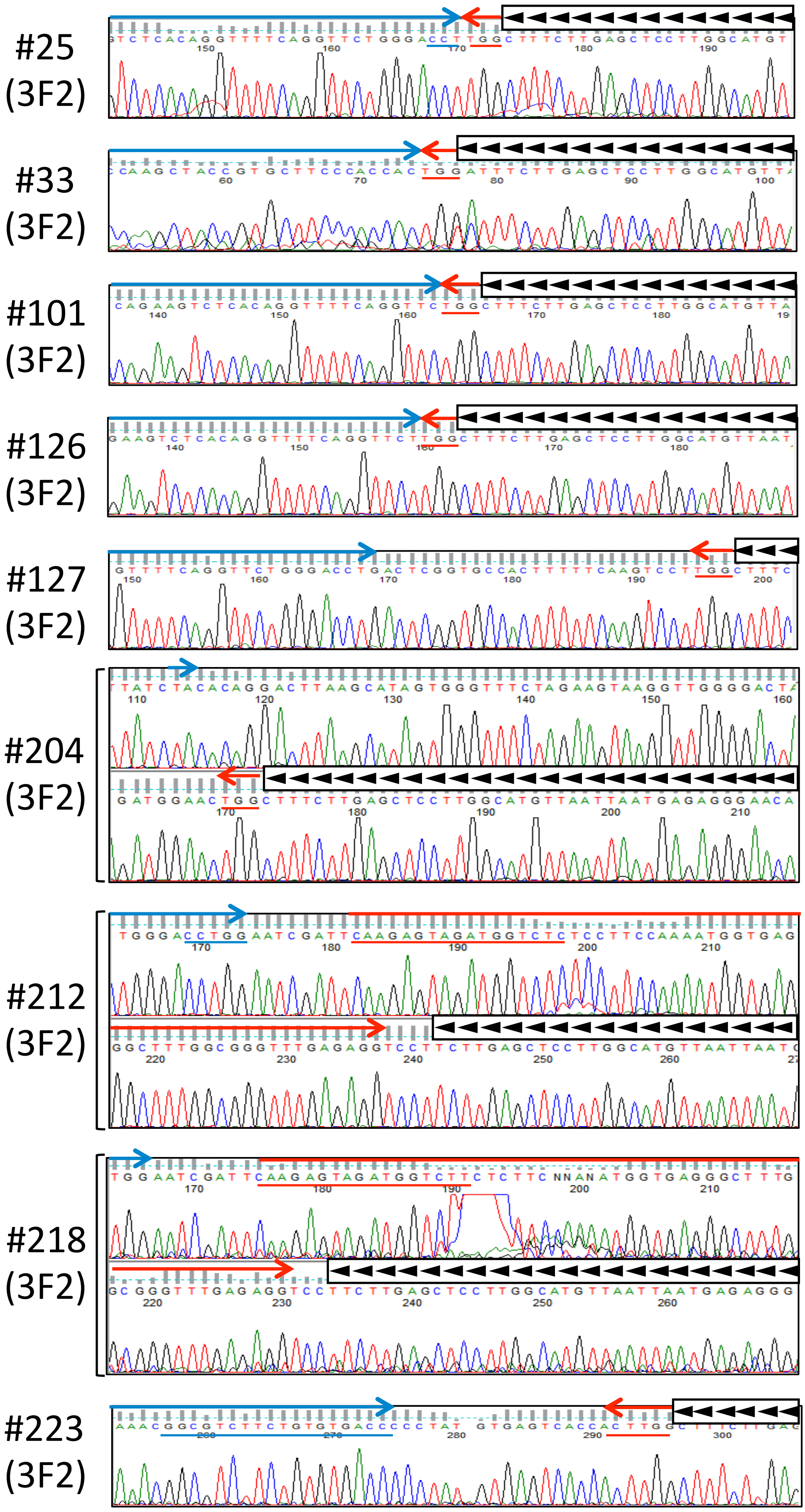

Supplementary Figure 3. continue

(3R1/1R2: detecting 3’ junction)

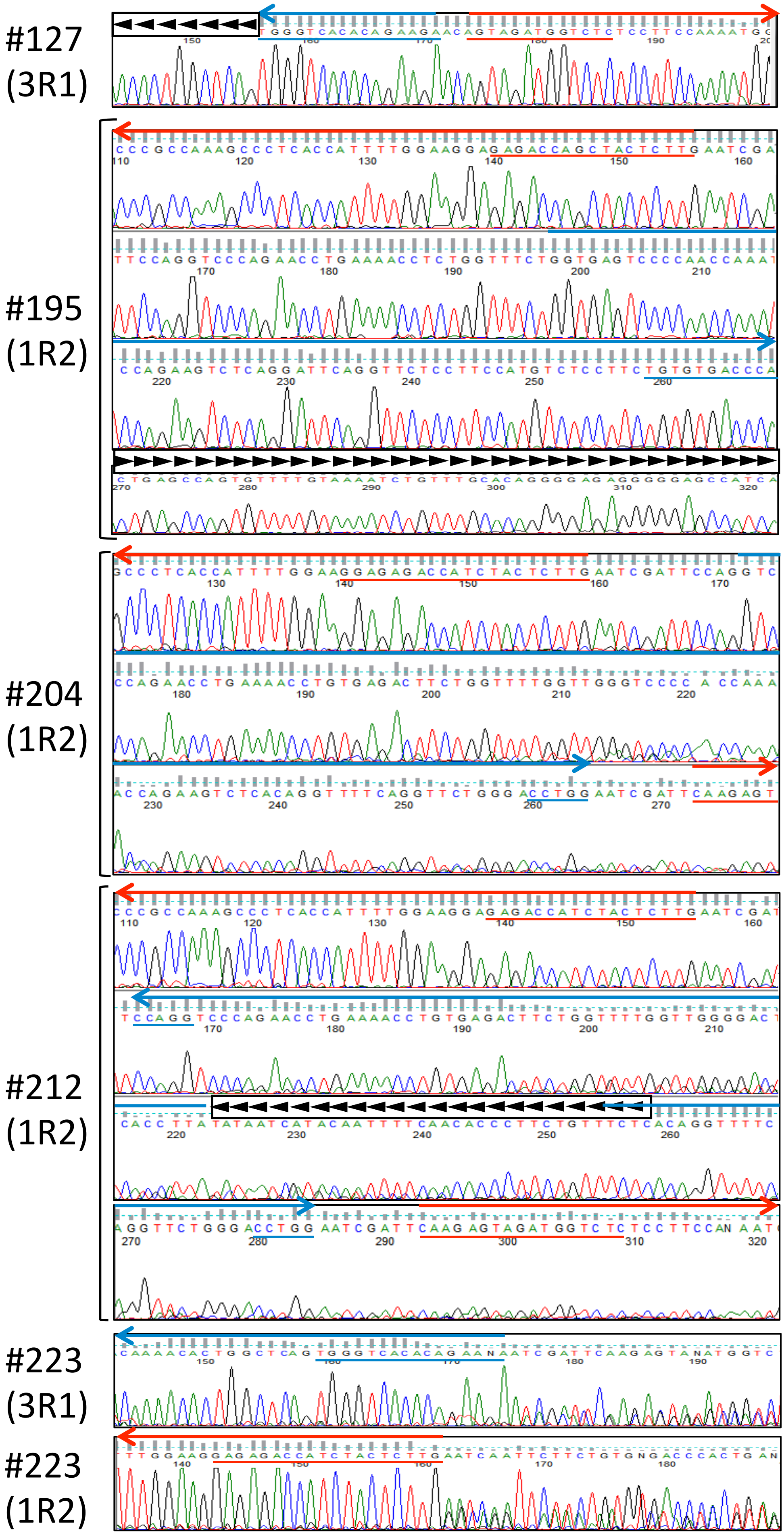

Supplementary Figure 3. continue

(3F3/1F2: detecting 3' junction)

#195
(3F2)

#195
(M13F)

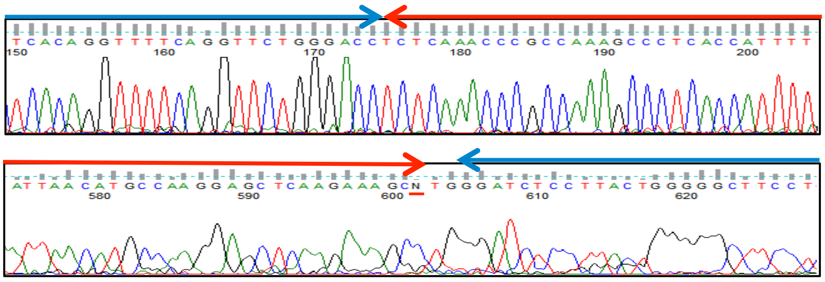

(3R3/1R4: detecting 3' junction)

#33
(3R1)

#218
(1R2)

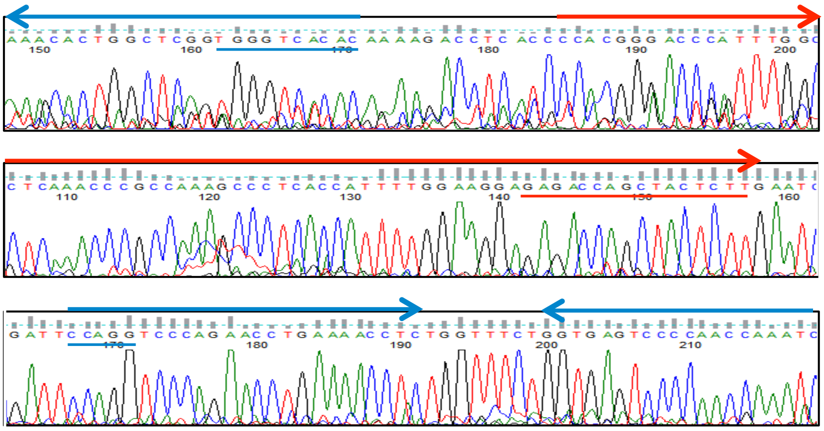

Supplementary Figure 3. Sequence of the 5 Mb of inversion junction .
An electrophoretogram of PCR products amplified using primers 3F1/1F1, 3R1/1R2, and 3R3/1R4. Note that arm3 and arm1 are indicated with blue and red arrows, respectively. sgRNA3 and sgRNA1 are shown with blue and red lines, respectively. The inverted region is indicated with a box containing arrowheads as shown in Figure 4A. 3F2, 3R1, and 1R2 in parentheses under the embryo ID represent the primers used for sequencing.

Supplementary Figure 4.

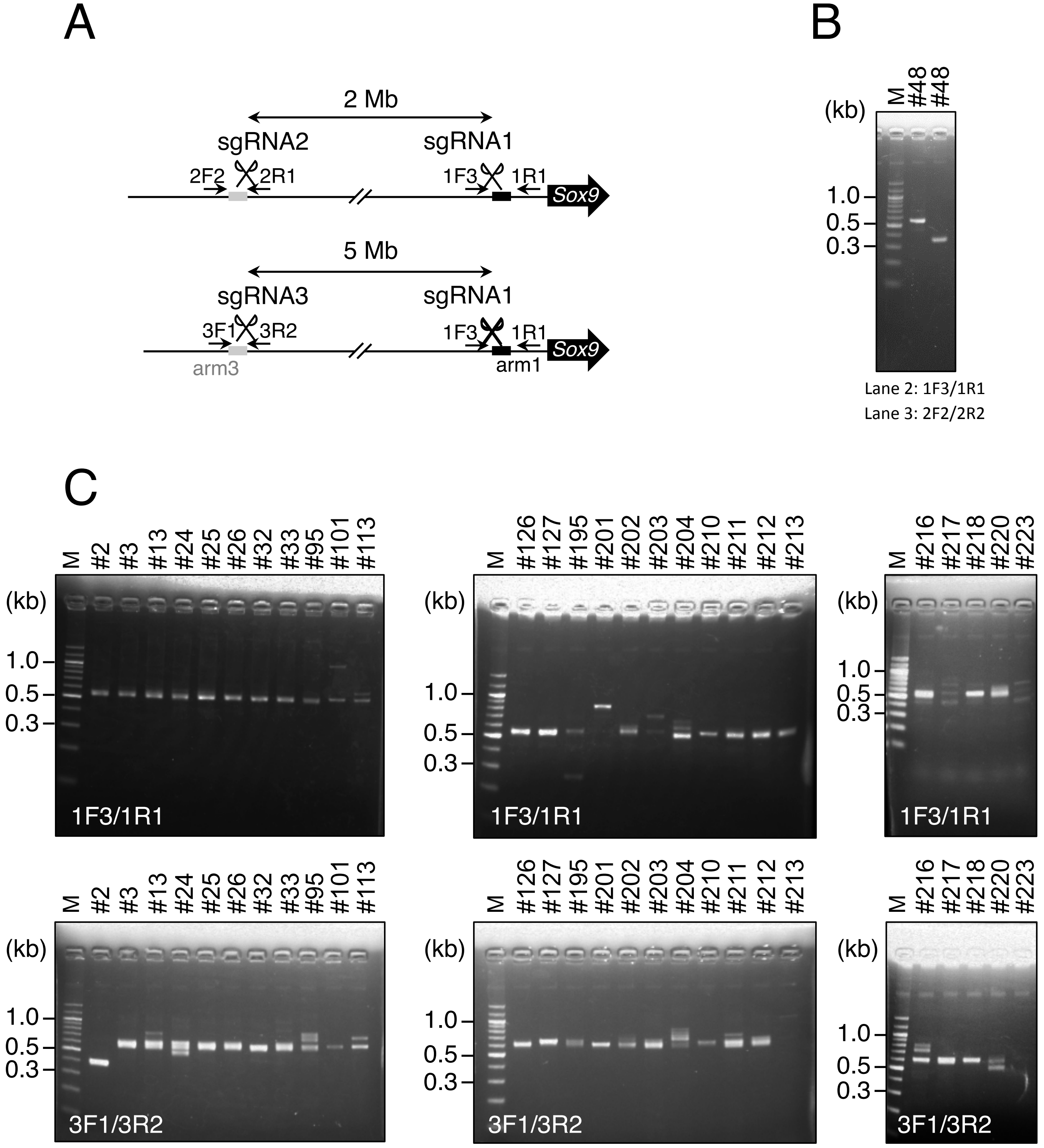

Supplementary Figure 4. Detection of the PCR amplicon surrounding sgRNAs of 2-Mb deletion and 5-Mb deletion or inversion mutant embryos

(A) Schematic representation of the 2-Mb and 5-Mb region upstream of Sox9. Black line represents a genome sequence. PCR primers are indicated with arrows. (B, C) An agarose gel electrophoretogram image of PCR products amplified using 1F3 and 1R1 primers (B), 2F2 and 2R2 primers (B), 1F3 and 1R1 primers (C), and 3F1 and 3R2 primers (C). DNA prepared from deletion or inversion embryos. Embryo IDs are shown above images with the # sign. M: a 1-kb DNA ladder (markers); N: no template control.

Supplementary Table 1. Oligomers used in this study.

Oligomer	Sequence (5'-3')
arm1FClaI	GACATCGATTCAAGAGTAGATGGTCTCTCC
arm1RHindIII	GACAAGCTTTGGAAGGGGAAGAAATGACTC
arm2FSalI	GACGTCGACTGGAAGGCTTCATGATCCTG
arm2RClaI	GACATCGATTATCTGGTTTCTTACAAACC
arm3FSalI	GACGTCGACCTGACTTGGATGCTCTGAAATCG
arm3RClaI	GACATCGATTCCAGGTCCCAGAACCTGAAAACC
sgRNA1F	AGACCATCTACTCTTGCCTGTTTTAGAGCTAGAAATAGCAAG
sgRNA1R	AACAGGCAAGAGTAGATGGTCTCGGTGTTTCGTCCTTTCCAC
sgRNA2F	AGATAATTACCATGCAACTGTTTTAGAGCTAGAAATAGCAAG
sgRNA2R	AACAGTTGCATGGTAATTATCTCGGTGTTTCGTCCTTTCCAC
sgRNA3F	GGGTCACACAGAAGACGCCGTTTTAGAGCTAGAAATAGCAAG
sgRNA3R	AACGGCGTCTTCTGTGTGACCCCGGTGTTTCGTCCTTTCCAC
T7sgRNA1F	TTAATACGACTCACTATAGGAGACCATCTACTCTTGCCT
T7sgRNA2F	TTAATACGACTCACTATAGGAGATAATTACCATGCAACT
T7sgRNA3F	TTAATACGACTCACTATAGGGGGTCACACAGAAGACGCC
1F1	AATGTTGCCCATCAGGTCTC
1F2	CGGAACTCCAACCATGTACC
1F3	TGAGAAGGGTGGTTCCAGTC
1R1	GAGTGTGAGGAAGGGATGGA
1R2	TCTGGACAGCTCTTCCACCT
1R3	AGGGACACAGGGCAATGTAG
1R4	TTGATCCTCCTTCTGGAACC
2F1	TGTCATGAATCAGGAATGCAA
2F2	CATTGATGCACTGCAATGAG
2R1	CCCATTTTGGTTCTCCTTCA
3F1	GATGGATGGGTGGTAGATGG
3F2	TGAGAAGGGTGGTTCCAGTC
3F3	AGGGAGACTCAAAGGGGAAA
3R1	ATTTGCTTCAGCGCTCCTTA
3R2	AGATATCTTGGGGCCACCTT
3R3	TCTGAGCATCCAGTGTGGAG
M13F	GTAAAACGACGGCCAGT
arm3-ClaI-arm1 ssODN	TAAGGTGAGTCCCCAACCAAAACCAGAAGTCTCACAGGTTTTCAGGTT CTGGGACCTGGAATCGATTCAAGAGTAGATGGTCTCTCCTTCCAAAAT GGTGAGGGCTTTGGCGGGTTTGAGAGGGTA

Recognition sequences of restriction endonucleases are underlined.
